# Occupation and cancer in Britain

**DOI:** 10.1038/sj.bjc.6605637

**Published:** 2010-04-27

**Authors:** L Rushton, S Bagga, R Bevan, T P Brown, J W Cherrie, P Holmes, L Fortunato, R Slack, M Van Tongeren, C Young, S J Hutchings

**Affiliations:** 1 Department of Epidemiology and Biostatistics, Faculty of Medicine, Imperial College London, St Mary's Campus, Norfolk Place, London W2 1PG, UK; 2 Institute of Environment and Health, Cranfield Health, Cranfield University, Cranfield, Bedfordshire MK43 0AL, UK; 3 Health and Safety Laboratory, Mathematical Sciences Unit, Harpur Hill, Buxton, Derbyshire SK17 9JN, UK; 4 Institute of Occupational Medicine, Research Avenue North, Riccarton, Edinburgh EH14 4AP, UK

**Keywords:** occupation, cancer burden, attributable fraction, industry sector, carcinogen

## Abstract

**Background:**

Prioritising control measures for occupationally related cancers should be evidence based. We estimated the current burden of cancer in Britain attributable to past occupational exposures for International Agency for Research on Cancer (IARC) group 1 (established) and 2A (probable) carcinogens.

**Methods:**

We calculated attributable fractions and numbers for cancer mortality and incidence using risk estimates from the literature and national data sources to estimate proportions exposed.

**Results:**

5.3% (8019) cancer deaths were attributable to occupation in 2005 (men, 8.2% (6362); women, 2.3% (1657)). Attributable incidence estimates are 13 679 (4.0%) cancer registrations (men, 10 063 (5.7%); women, 3616 (2.2%)). Occupational attributable fractions are over 2% for mesothelioma, sinonasal, lung, nasopharynx, breast, non-melanoma skin cancer, bladder, oesophagus, soft tissue sarcoma, larynx and stomach cancers. Asbestos, shift work, mineral oils, solar radiation, silica, diesel engine exhaust, coal tars and pitches, occupation as a painter or welder, dioxins, environmental tobacco smoke, radon, tetrachloroethylene, arsenic and strong inorganic mists each contribute 100 or more registrations. Industries and occupations with high cancer registrations include construction, metal working, personal and household services, mining, land transport, printing/publishing, retail/hotels/restaurants, public administration/defence, farming and several manufacturing sectors. 56% of cancer registrations in men are attributable to work in the construction industry (mainly mesotheliomas, lung, stomach, bladder and non-melanoma skin cancers) and 54% of cancer registrations in women are attributable to shift work (breast cancer).

**Conclusion:**

This project is the first to quantify in detail the burden of cancer and mortality due to occupation specifically for Britain. It highlights the impact of occupational exposures, together with the occupational circumstances and industrial areas where exposures to carcinogenic agents occurred in the past, on population cancer morbidity and mortality; this can be compared with the impact of other causes of cancer. Risk reduction strategies should focus on those workplaces where such exposures are still occurring.

Reduction of occupationally related cancers requires sound evidence on which to base priorities. Recognition and classification of a substance as a carcinogen is made through scrutiny and assessment of a wide range of evidence including *in vivo, in vitro* and human studies. For substances that are already established as human carcinogens, for example using the International Agency for Research on Cancer (IARC) classification, estimation of attributable burden provides useful indicators of the contribution of different risk factors and has become widely used in public health research (Lopez *et al*, 2006). Risk reduction can thus incorporate consideration of risk level with the size of potentially exposed populations. A comprehensive review of the proportions of cancer from different causes in the United States estimated, the contribution of occupational factors as 4% with an uncertainty range of 2–8% (Doll and Peto, 1981). These estimates have been used to formulate occupational health policies in many countries, including Britain. More recently Doll and Peto (2005) have also produced an estimate for Britain of 2% with a range of 1–5% they suggested that less than 1% is avoidable by practicable ways (Doll and Peto, 2005). Budget rationing based on underestimates of the cancer burden attributed to occupation will almost certainly overlook important issues. Conversely, overestimation of the cancer burden may result in tighter regulation, which may impede industry. Therefore, it is important to develop rigorous methodology to estimate cancer burden, which is adaptable to a given country.

The attributable fraction (AF) is widely used, for example, in the estimation of global burden of disease (Driscoll *et al*, 2005); quantification and ranking of the burden by diseases and causes of disease facilitate decision making for risk reduction measures. Our paper presents an evaluation of the burden of cancer in Britain for all carcinogenic agents and occupations classified by IARC as a group 1 (established) or 2A (probable) carcinogen that, for occupational exposures, had either ‘strong’ or ‘suggestive’ evidence of carcinogenicity in humans for the specific cancer site, as defined by Siemiatycki *et al* (2004) and subsequent IARC publications (Rousseau *et al*, 2005; Straif *et al*, 2005, 2007). We identify carcinogens, occupations and industry sectors that make an important contribution to the total burden.

## Materials and methods

Preliminary results for six cancer sites, showing the developing methodology have been previously published (Rushton *et al*, 2008). For this paper, estimations were carried out using 2005 data for mortality and 2004 for cancer incidence. Mortality data were obtained from the Office for National Statistics (ONS), and the General Register Office for Scotland. Cancer incidence data were obtained from ONS, Cancer Statistics, Registrations, Series MB1 for England, the Scottish Cancer Registry, and the Welsh Cancer Intelligence and Surveillance Unit.

We estimated the AF, that is, the proportion of cases that would not have occurred in the absence of an occupational exposure; this was then used to estimate the attributable numbers (ANs). There are several methods for estimating the AF but all depend on knowledge of the disease risk due to the exposure of interest and the proportion of the target population exposed (Steenland and Armstrong, 2006).

Risk estimates were obtained from key studies, meta-analyses or pooled studies, taking into account quality (including relevance to Britain, sample size, extent of control for confounders, adequacy of exposure assessment and clarity of case definition). Where possible we selected risk estimates adjusted for important confounders or non-occupational risk factors, for example, smoking for lung cancer, smoking and alcohol use for laryngeal cancer. Where only a narrative review was available giving a range of risk estimates, we calculated a combined estimate of the relative risks (RRs) based on a random-effects (for heterogeneous RRs) or fixed-effects (for homogeneous RRs) model. Formal systematic reviews and meta-analyses were carried out to estimate risk estimates for laryngeal and stomach cancers related to asbestos exposure.

Dose–response risk estimates were generally not available in the epidemiological literature nor were proportions of those exposed at different levels of exposure over time available for the working population in Britain. However, where possible risk estimates were obtained for an overall ‘lower’ level and an overall ‘higher’ level of exposure to the agents of concern. The risk estimates for occupational exposure to ionising radiation were derived using generalised linear dose–response models of excess RR per unit of cumulative radiation dose from the United Nations Scientific Committee on the Effects of Atomic Radiation (UNSCEAR, 2006). Cumulative lifetime dose was estimated using data from the Central Index of Dose Information (CIDI) (HSE, 1998). For aircrew, who are not covered by CIDI, the mean total lifetime radiation dose per pilot was obtained from a large cohort study of European airline pilots (Langner *et al*, 2004) and combined with numbers employed obtained from the British Airways Stewards and Stewardesses Union (BASSA, personal communication, 2008).

A substantial proportion of the excess is likely to occur in the large number of workers with low exposures for whom our estimates of average risks are inevitably unreliable. Where no risk estimate could be identified for very low or background levels of exposure, we estimated an RR for the ‘lower exposed’ group by (1) taking the harmonic mean of all the available ratios of ‘higher’ to ‘lower’ RR estimates for cancer-exposure pairs for which data were available and (2) applying this average ratio to the ‘higher’ level estimates to obtain ‘lower’ level RR estimates; if this was less than one, it was set to one. (In the developmental phase of the study, an RR of one was arbitrarily assigned, giving zero AF and so possibly underestimating the burden; a large number of people exposed at low levels associated with a low risk of disease may contribute more to the burden than a small number exposed at high levels associated with a high risk.)

We defined the period during which the relevant exposure for the cancer in the target year 2005 as the risk exposure period (REP). For solid tumours, a latency of 10–50 years was assumed giving an REP of 1956–1995; for haematopoietic neoplasms, 0–20 year's latency was assumed giving an REP of 1986–2005. The proportion of the population ever exposed to each carcinogenic agent or occupation in the REP was obtained from the ratio of the numbers ever exposed to the carcinogens of interest in each relevant industry or occupation within Britain over the total number of people ever employed (Equation (4) in the Statistical Appendix).

If the study from which the risk estimates were obtained was population based, an estimate of the proportion of the population exposed was derived directly from the study data, although such studies were rarely available for Britain. If the risk estimate was obtained from an industry-based study, national data sources such as the CARcinogen EXposure (CAREX) database (Pannett *et al*, 1998), the UK Labour Force Survey (LFS) (LFS, 2009) and the Census of Employment (ONS, 2009) were used. CAREX was used for estimating the numbers of the British population ever exposed to a carcinogen by industry sector. As highlighted above, data are not available on the levels of exposure in all industry sectors for all the carcinogens considered, nor the numbers exposed at these levels. Industry sectors were therefore allocated to ‘higher’ or ‘lower’ exposure categories assuming distributions of exposure and risk that corresponded broadly to those of the studies from which the risk estimates were selected. The initial allocations were based on the judgement of an experienced human exposure scientist; each assessment was then independently peer reviewed and if necessary, a consensus assessment agreed. Data from CAREX are not differentiated by sex; 1991 Census data by industry and occupation were used to estimate the relative proportions of men and women exposed. The LFS and Census of Employment data were used to estimate numbers ever employed in specific occupations, for example, welder, painter and so on, and for specific industries for carcinogens not included in CAREX.

CAREX data for Britain relate only to the period 1990–1993. For the LFS and CoE, an available year was chosen to represent numbers employed about 35 years before the target year of 2005, as this was thought to represent a ‘peak’ latency for the solid tumours, and is also close to the mid-point of the REP for estimating numbers ever exposed across the period (for which a linear change in employment levels was implicitly assumed). Where the Census of Employment was used, the data are for 1971. Where the LFS was used, the first year available and therefore used was 1979 for solid tumours, and 1991 for short latency cancers. When CAREX data were used, adjustment factors were applied to take account of the change in numbers employed in the primary and manufacturing industry and service sectors in Britain over the REP. Adjustment for employment turnover over the period for grouped main industry sectors was also carried out (see Equation (3) in the Statistical Appendix). Ideally this requires full national starter and leaver data across the REP for all industry sectors. In the absence of this quality of data, estimating turnover directly using new starters in years within the REPs gives the best approximation for the purpose of estimating those ever exposed. This method estimates starters in the past year as a proportion of the average number employed (Gregg and Wadsworth, 2002). As exposure in occupational epidemiological studies is usually defined as for at least 1 year, we have adapted this to exclude short-term labour turnover by taking new starters in the past year who are expected to remain employed for at least 1 year as a proportion of all those expected to be employed for at least 1 year. This is estimated as the number recorded as employed for between 1 and 2 years divided by the total employed for at least 1 year using LFS data averaged over the REP.

### Statistical analysis

To estimate the AFs for each cancer and occupational carcinogen, we used Levin's method if risk estimates came from an industry-based study, a review or meta-analysis, together with estimates of the proportion of the population exposed from independent national sources of data (Levin, 1953). Miettinen's method was used if risk estimates and proportions exposed came from a population-based study (Miettinen, 1974) (Equations (1) and (2), respectively, in the Statistical Appendix). For each AF, a random error confidence interval was calculated using Monte Carlo simulations (Steenland and Armstrong, 2006). The AFs were applied to total numbers of cancer-specific deaths (2005) and cancer registrations (2004) for ages that could have been exposed during the REP to give ANs. Where risk estimates were only available from mortality studies, AFs derived from these were used for estimation of attributable registrations and vice versa. Similarly if separate AFs for women could not be estimated, those for men or for men and women combined were used.

The AF for mesothelioma was derived directly from several UK mesothelioma studies that suggest 96–98% of male mesothelioma cases are due to occupational or paraoccupational (e.g., exposure from living near an asbestos factory or handling clothes contaminated due to occupational exposure) exposure (Howel *et al*, 1997; Yates *et al*, 1997; Rake *et al*, 2009). Combining the results from Rake *et al* (2009) with those from two studies in which results were reported separately for females (Spirtas *et al*, 1994; Goldberg *et al*, 2006) gave estimates of 75–90% for females. The ratio of asbestos-related lung cancer to mesothelioma deaths has been suggested to be between two-thirds and one (Darnton *et al*, 2006). Rather than our standard method for the estimation of numbers of lung cancers attributable to asbestos, we therefore used a ratio of 1 : 1 mesothelioma to lung cancer deaths; this takes into account the impact that past levels of asbestos exposure are having on current incidence by the direct link to mesothelioma deaths that are still climbing whereas lung cancer in general is declining due to the reduction in smoking. This assumes, however, that lung cancer has a similar pattern of latency as mesothelioma. For lung cancer associated with radon exposure from natural sources, estimates of rates of lung cancer due to exposure to radon in domestic buildings (NRPB, 2000) were applied to estimates of the time employees spend in workplaces where radon exposure occurs.

AFs for all the relevant carcinogenic agents and occupational circumstances were combined into a single estimate of AF for each separate cancer. To take account of potential multiple exposures, we used strategies including partitioning exposed numbers between overlapping exposures or estimating only for the ‘dominant’ carcinogen with the highest risk. The IARC Monograph process has been taking place over many years and has resulted in overlap between substances evaluated. For lung cancer, for example, 32 occupations or carcinogenic agents are evaluated. We estimated AFs for 21 of these; for example, substances such as coal tars and pitches and processes such as coal gasification and coke production were included within our evaluation of polycyclic aromatic hydrocarbons (PAHs). Where exposure to multiple carcinogens remained, it was assumed that the exposures were independent of one another and that their joint carcinogenic effects were multiplicative. The AFs were then combined to give an overall AF for that cancer using a product sum (Equation (5) in the Statistical Appendix). An overall AF for all cancers was estimated by summing the ANs for each and dividing by the total number of cancers in Britain.

## Results

The overall burden by cancer site (AFs, ANs and 95% confidence intervals) is given in Table 1. In all, 8.2% (*n*=6362) of cancer deaths in 2005 in men and 2.3% (*n*=1657) in women in Britain have been estimated to be due to occupation giving an overall AF of 5.3% (*n*=8019). The combined AFs for registrations are 5.7% (*n*=10 063) for men in 2004 and 2.2% (*n*=3616) for women giving an overall AF based on registrations of 4.0% (*n*=13 679). These are lower than for deaths because of the large numbers of non-melanoma skin cancer (NMSC). The results for four of the cancers, bladder, leukaemia, NMSC and sinonasal, are lower than previously estimated (Rushton *et al*, 2008) mainly due to reallocation of some of the industry sectors from ‘higher’ to ‘lower’ exposure categories after more in-depth review of the exposures in Britain. If only agents and occupations classified by IARC as group 1 and having strong evidence of carcinogenicity in humans are considered, the overall burden reduces to 4.0% (5123 total deaths, 8277 total registrations) (Supplementary Table A1). Only nine cancer sites are involved (bladder, larynx, leukaemia, liver, lung, mesothelioma, NMSC, sinonasal and thyroid). The dominance of asbestos exposure and mesothelioma, asbestos and the many other group 1 carcinogens relevant to lung cancer, and solar radiation (SR) and NMSC means that the reduction in the AFs and ANs for men (6.6%, 5123 deaths, 8277 registrations) is far less than for women (1.2%, 862 deaths, 1313 registrations) for whom shift work is most dominant.

The AFs by cancer site range from less than 0.01–95% overall, the most important cancer sites for occupational attribution being, for men, mesothelioma (97%), sinonasal (46%), lung (21.1%), bladder (7.1%) and NMSC (7.1%), and for women, mesothelioma (83%), sinonasal (20.1%), lung (5.3%), breast (4.6%) and nasopharynx (2.5%). Occupation also contributes 2% or more overall to cancers of the larynx, oesophagus, soft tissue sarcoma (STS) and stomach, with in addition for men melanoma of the eye (due to welding) and non-Hodgkin's lymphoma (NHL).

Table 2 gives the number of registrations in 2004 by cancer site for each cancer attributable to each of the 41 agents and occupations for which separate estimation was carried out, considered in rank order. Of these, 15 contributed over 100 total cancer registrations, the largest being asbestos exposure (mesothelioma (1937), lung (2223), larynx (8) and stomach cancers (47)), followed in order by shift work, including flight personnel (breast (1969), mineral oils (bladder (296), lung (470), NMSC (902), sinonasal (63)), SR (NMSC (1541)), silica (lung (907)), diesel engine exhaust (DEE) (lung (695), bladder (106)), PAHs from coal tar and pitches (NMSC (545)), occupation as a painter (bladder (71), lung (282), stomach (5)), dioxins (lung (215), NHL (74), STS (27)), environmental tobacco smoke (ETS) at work in non-smokers (lung (284)), radon exposure from natural exposure in workplaces (lung (209)), occupation as a welder (lung (175), and melanoma of the eye due to UV radiation (6)), tetrachloroethylene (cervix (18), NHL (17), oesophagus (130)), arsenic (lung (129)) and strong inorganic acid mists (larynx (46), lung (76)). The results in this table highlight the fact that many carcinogenic exposures in the workplace can affect multiple cancer sites.

Table 3 gives numbers of registrations within industry sectors or occupations for which there were at least 50 total attributable registrations. The exposures concerned in each industry sector are listed, with those contributing most (at least a total of 10 cancers) being shown in bold. Painters and welders are assumed to be exposed to many different carcinogens. Also given are the relevant cancer sites for each industry sector with the most important highlighted in bold (at least a total of 10 cancers). Of a total of 60 industry sectors, 21 have 100 or more total attributable registrations. The majority of industry sectors involve exposure to several carcinogens (many over 10) with construction and many of the manufacturing sectors involving potential exposure to between 15 and 20. In addition, the potential occurrence of several exposures in what might be thought of as less traditionally exposed sectors, such as the retail and service industries, is highlighted. There are several key exposures that give rise to substantial numbers of registrations across multiple cancer sites and multiple industry sectors. Of note is the contribution of exposure to (1) asbestos, DEE, silica and SR in the construction industry; (2) asbestos, DEE, ETS (non-smokers), soots and tetrachloroethylene in personal and household services (this sector includes repair trades, laundries and dry cleaning, domestic services, hairdressing and beauty); (3) asbestos and DEE in land transport (railway, road and pipeline); (4) asbestos, DEE, silica and SR in mining; (5) ETS (non-smokers) and SR in public administration and defence; (6) asbestos, ETS (non-smokers) and radon in the wholesale and retail trade, restaurants and hotels; and (7) dioxins, non-arsenical pesticides and SR in farming.

## Discussion

Our study has quantified for the first time the impact of occupation on the burden of cancer in Britain for all 24 cancer sites and the carcinogens that IARC have classified as having sufficient (group 1) or limited (group 2A) evidence in humans. Unlike other estimates of cancer burden, we have also evaluated the impact within different industry sectors, so facilitating prioritisation and implementation of preventive action. Our results are greater than other estimates for some cancer sites, partly due to differences in the numbers of cancers and carcinogens considered. For example, the global estimate of occupational cancer (Driscoll *et al*, 2005) included only lung cancer, leukaemia and mesothelioma; their estimate for lung cancer included only eight carcinogens, arsenic, asbestos, beryllium, cadmium, chromium, DEE, nickel and silica. The 13 additional agents in our study contributed 34% of the total attributable registrations from lung cancer. The relative importance of different cancer sites also changes as more cancer sites are included, with leukaemia (included in the global estimate) being less important in our study than other sites, including other haematopoietic malignancies such as NHL.

Our methodology was developed with advice, discussion and peer review from international experts, including IARC, throughout the project and at two international workshops. It takes account of issues such as latency and the period in which relevant exposure would occur, changes in workforce turnover and employment trends and the potential for concurrent exposure to several carcinogens and at different levels. These methods have the potential to be adapted for use in other countries and extended to include social and economic impact evaluation. For example, both the methods and results from the study are currently being used to inform an EU project to assess the socioeconomic impact of, and make recommendations for, new occupational exposure limits for the European Union for 25 recognised carcinogens. It is also being used to underpin the on-going update of the World Health Organisation Global Burden of Disease.

However, assumptions made in our methodology and uncertainties and inaccuracies in our data may have introduced biases into our estimates, the impact of which is not fully captured in the confidence intervals presented. Potential sources of bias include inappropriate choice of risk estimates, imprecision in the risk estimates and estimates of proportions exposed, inaccurate REP and latency assumptions and a lack of separate risk estimates in some cases for women and/or cancer incidence. Future work will explore the sensitivity of the estimates to such sources of uncertainty and bias. Our estimates do not include evaluation of the results from an on-going review and update of all group 1 carcinogens, which IARC is carrying out separately for cancer sites. This classification can vary for the same substance; thus, asbestos is classified as group 1 for mesothelioma and cancers of the larynx, lung and ovary and as group 2A for colorectal, pharyngeal and stomach cancers (Straif *et al*, 2009).

Our estimate of the fraction of current cancer deaths in Britain due to past occupational exposures is at the upper limit of the range of 1–5% suggested by Doll and Peto (2005). The earlier and much higher estimate for the United States in the unpublished but widely cited report by Bridbord *et al* (1978) was criticised by Doll and Peto (1981) for disregarding both dose and duration of exposure and assuming that all workers (including some unexposed) were at equally high risk. The ideal requirement would be quantitative exposure estimates in Britain for each of the relevant cancers and carcinogens; unfortunately, these are rarely available. However, we have been careful to use estimates of RR from studies that were conducted in populations where exposure levels are representative of the level of risk to which British workers are likely to have been exposed. The allocation of industries included in CAREX to ‘higher’ or ‘lower’ exposure level categories was also based on the levels of exposure described in the study from which the risk estimates were drawn. Where CAREX data were not available, estimates of numbers employed were obtained from the LFS or Census of Employment; job or industry categories were chosen that conformed as nearly as possible those in the studies from which the RRs were drawn. In addition, the risk estimates from the literature usually related to an estimate of cumulative exposure. In assigning ‘higher’ and ‘lower’ categories to the CAREX industry groups, implicit assumptions were made regarding the similarity of durations and intensities of exposure between the source and target (national) populations. National data are not generally available on the proportions of those exposed at different levels of exposure, nor does the United Kingdom have a national job exposure matrix such as those developed in Finland and the United States (Antilla *et al*, 1992; Greife *et al*, 1995).

Due to the long latent interval of many carcinogens, our estimates of current burden are based on past exposures, many of which would have been considerably higher than today; there is evidence of continuing downward trends in the United Kingdom in many exposures (Cherrie *et al*, 2007). A comprehensive analysis of published exposure data, particularly in Western Europe and North America, has shown similar patterns of decreasing exposure patterns across a wide range of industries and substances (Symanski *et al*, 1998a, 1998b). It should be noted that for many of the carcinogens in our study a major contribution to the burden was made by a large number of workers exposed at low levels and low risk for which our quantitative risk estimates are inevitably uncertain. Our study has highlighted the fact that many workers may potentially be exposed to several carcinogens and that these may affect multiple cancer sites. Key carcinogens and industry sectors have been identified posing varying challenges for risk reduction strategies; these include asbestos, DEE, ETS (non-smokers), radon, silica, SR and shift work.

Substantial exposure to asbestos in Britain until the 1970s has contributed to a continuing rise in asbestos-related deaths from lung cancer and mesothelioma (Hodgson *et al*, 2005); asbestos contributes 30% of the occupationally related cancer registrations and nearly half of the deaths in our study. This highlights exposure in the construction industry along with other dusts such as those containing silica. A recent case–control study of UK mesothelioma patients found high risks associated with asbestos exposure in the construction industry, including work as a plumber, electrician, painter or decorator Rake *et al* (2009). It was suggested that a substantial proportion of the deaths in carpenters would be among those who installed asbestos installation board as fireproofing; the large amount of asbestos that remains in many older buildings may still be a potential hazard to construction workers involved in renovation, maintenance or asbestos removal. Difficulties of managing asbestos exposures arising during industrial maintenance and repair and the problems of health protection of workers worldwide have been reported (Wagner, 2007). Exposure to dusts containing silica is also highlighted in several industry sectors in our study. Measurements of respirable crystalline silica in the UK construction industry have found levels that greatly exceed the current maximum exposure limit of 0.3 mg m^−3^, particularly for work in confined spaces and without water suppression of dust or effective extraction (Chisholm, 1999).

Diesel engines have a wide range of industrial applications including on-road equipment (most heavy and medium duty trucks and buses use diesel engines), and off-road applications in the mining, rail, construction, distribution and farming industries and in the military, including the use of diesel-powered heavy equipment, locomotives, forklift trucks, ships, tractors and generators. In a recent review, the highest levels of elemental carbon (EC) were reported for enclosed underground work sites in mining and construction (EC 27–658 *μ*g m^−3^) (Pronk *et al*, 2009). Intermediate levels were reported for above-ground semi-enclosed areas involving work as a workshop mechanic, dock worker and fire station workers (EC<50 *μ*g m^−3^), with the lowest levels (EC<25 *μ*g m^−3^) reported for enclosed areas separated from the source, such as drivers, train crew, parking attendants, vehicle testers and utility service workers.

Cancer in non-smokers due to workplace exposure to ETS may be expected to largely disappear in Britain in future due to the ban now in force; our results imply the importance of this exposure in countries where no ban is yet enforced. Carcinogens such as naturally occurring radon could also easily be eliminated from workplaces. Our study has extended evaluation of more ‘traditional’ industrial hazards such as asbestos to estimation of the effect of newer and less traditional problems. In 2007 IARC classified shift work, in particular night work, as being group 2A for breast cancer (Straif *et al*, 2007). The estimate of nearly 2000 breast cancer registrations due to shift work in our study is 54% of all female occupationally related cancer registrations (1969/3616). The epidemiological evidence evaluated by IARC comes from population-based case–control studies and studies of nurses, marine telephone operators and female flight attendants, although in the latter the effects of concomitant exposure to shift work and ionising radiation is difficult to disentangle. Large studies in other industry groups with collection of good shift pattern data are needed. Denmark recently became the first country to designate breast cancer as an occupational disease eligible for receiving compensation (Commentary, 2009). The ramifications of this decision and of our results could be significant, given the large numbers of women working night shifts in Britain and worldwide.

Exposure to SR and mineral oils at work was associated with a large number of registrations of NMSC (21% of all attributable registrations). Mortality from NMSC is low but there may be appreciable morbidity from disfigurement as the lesions tend to be on the head and neck; their high prevalence represents a considerable health service burden (Houseman *et al*, 2003; Boyle *et al*, 2004; Trakatelli *et al*, 2007). As NMSC is thought to be substantially underregistered, our occupational figures are also likely to be underestimated.

The ANs and AFs presented in this paper provide a basis on which to inform prioritisation of future effort both for health and safety strategic planning and for research to fill information gaps. Reduction and removal of these carcinogens might be expected to increase average life expectancy. Other measures such as years of life lost and disability-adjusted life years will be estimated as part of our on-going study.

In summary, this project is the first to quantify in detail the burden of cancer due to occupation specifically for Britain. It highlights the impact of such exposures on cancer morbidity and mortality, together with the occupational circumstances and industrial areas where exposures to these agents occurred in the past; this can be compared with the impact of other causes of cancer. Risk reduction strategies should focus on those workplaces where such exposures are still occurring.

## Figures and Tables

**Table 1:** Estimated attributable fractions, deaths and registrations by cancer site in 2005 (deaths) and 2004 (registrations)

		**Attributable fraction (%) (95% confidence interval)**			**Attributable numbers (95% confidence interval)**					
					**Deaths (2005)**			**Registrations (2004)**		
**Cancer site**	**ICD-10 code**	**Male**	**Female**	**Total (based on deaths)**	**Male**	**Female**	**Total**	**Male**	**Female**	**Total**
Bladder	C67	7.1 (4.6, 9.7)	1.9 (1.3, 3.9)	5.3 (3.4, 7.7)	215 (139, 296)	30 (21, 62)	245 (159, 358)	496 (321, 684)	54 (37, 110)	550 (357, 795)
Bone	C40–C41	0.04	0.01	0.02	0	0	0	0	0	0
Brain	C70–C72	0.5 (0.1, 1.1)	0.1 (0, 0.2)	0.4 (0.0, 0.7)	10 (1, 20)	1 (0, 3)	11 (1, 23)	12 (1, 25)	2 (0, 4)	14 (1, 28)
Breast	C50		4.6 (3.3, 6.0)	4.6 (3.3, 6.0)		555 (397, 727)	555 (397, 727)		1969 (1407, 2579)	1969 (1407, 2579)
Cervix	C53		0.7 (0.0, 2.1)	0.7 (0.0, 2.1)		7 (0, 22)	7 (0, 22)		18 (1, 56)	18 (1, 56)
Kidney	C64–C66, C68	0.04 (0, 0.16)	0.04 (0, 0.14)	0.04 (0, 0.15)	1 (0, 3)	1 (0, 2)	1 (0, 5)	2 (0, 7)	1 (0, 4)	3 (0, 10)
Larynx	C32	2.9 (1.4, 5.7)	1.6 (0.6, 3.5)	2.6 (1.2, 5.2)	17 (8, 34)	3 (1, 6)	20 (9, 40)	50 (24, 99)	6 (2, 13)	56 (26, 112)
Leukaemia^a^	C91–C95	0.9 (0.2, 3.5)	0.5 (0.1, 4.5)	0.8 (0.2, 3.9)	18 (4, 71)	6 (1, 49)	24 (5, 120)	31 (7, 118)	9 (1, 80)	40 (8, 199)
Liver	C22	0.2 (0.1, 0.3)	0.1 (0.1, 0.2)	0.2 (0.1, 0.3)	4 (2, 6)	2 (1, 2)	5 (3, 8)	4 (2, 6)	1 (1, 2)	5 (3, 8)
Lung	C33–C34	21.1 (19.2, 24.7)	5.3 (4.3, 6.9)	14.5 (13.0, 17.2)	4024 (3659, 4696)	726 (592, 946)	4749 (4251, 5643)	4632 (4212, 5406)	816 (666, 1063)	5447 (4877, 6469)
Lympho-haematopoietic	C81–C96	0.004 (0, 0.014)	0.002 (0, 0.007)	0.003 (0, 0.011)	0 (0, 1)	0 (0, 0)	0 (0, 1)	0 (0, 1)	0 (0, 0)	1 (0, 2)
Melanoma (eye)	C69	2.9 (0.6, 6.6)	0.4 (0.1, 1.0)	1.6 (0.3, 3.6)	1 (0, 3)	0 (0, 0)	1 (0, 3)	6 (1, 13)	1 (0, 2)	6 (1, 16)
Mesothelioma	C45	97.0 (96.0, 98.0)^b^	82.5 (75.0, 90.0)^b^	94.9 (93.0, 96.9)^b^	1699 (1681, 1717)	238 (216, 260)	1937 (1898, 1976)	1699 (1681, 1717)^c^	238 (216, 260)^c^	1937 (1898, 1976)^c^
Multiple myeloma	C90	0.4 (0, 1.0)	0.1 (0, 0.3)	0.3 (0, 0.7)	5 (0, 10)	1 (0, 2)	6 (0, 12)	8 (0, 18)	2 (0, 3)	10 (0, 21)
Nasopharynx	C11	11.0 (2.3, 47.9)	2.5 (0.6, 6.8)	8.2 (1.8, 34.3)	7 (2, 32)	1 (0, 2)	8 (2, 33)	14 (3, 61)	2 (0, 4)	16 (3, 65)
NHL	C82–C85	2.1 (0, 6.9)	1.1 (0.1, 2.9)	1.7 (0, 5.4)	43 (0, 138)	14 (1, 37)	57 (1, 176)	102 (0, 328)	39 (3, 101)	140 (3, 430)
NMSC^d^	C44	7.1 (1.3, 15.1)	1.1 (0.0, 2.9)	4.6 (0.8, 10.0)	21 (4, 44)	2 (0, 6)	23 (4, 50)	2576 (481, 5475)	352 (0, 900)	2928 (481, 6375)
Oesophagus	C15	3.3 (1.5, 7.5)	1.1 (0.3, 2.8)	2.5 (1.1, 5.9)	156 (70, 358)	28 (8, 70)	184 (78, 429)	159 (71, 365)	29 (9, 74)	188 (80, 439)
Ovary	C56		0.5 (0, 1.2)	0.5 (0, 1.2)		23 (0, 52)	23 (0, 52)		33 (0, 76)	33 (0, 76)
Pancreas	C25	0.02 (0, 0.07)	0.01 (0, 0.04)	0.01 (0, 0.05)	1 (0, 2)	0 (0, 1)	1 (0, 4)	1 (0, 2)	0 (0, 1)	1 (0, 4)
Sinonasal	C30–C31	46.0 (27.3, 74.0)	20.1 (14.4, 31.6)	34.4 (21.5, 54.8)	29 (17, 47)	10 (8, 16)	39 (25, 63)	101 (60, 162)	32 (23, 50)	133 (83, 212)
STS	C49	3.4 (0, 11.4)	1.1 (0, 3.8)	2.4 (0, 8.1)	11 (0, 36)	3 (0, 9)	13 (0, 45)	22 (0, 75)	4 (0, 15)	27 (0, 90)
Stomach	C16	3.0 (1.5, 5.1)	0.3 (0.1, 0.5)	2.0 (1.0, 3.4)	102 (52, 176)	6 (3, 11)	108 (55, 187)	149 (77, 258)	9 (4, 15)	158 (81, 274)
Thyroid	C73	0.12	0.02	0.05	0	0	0	1	0	1
Total based on deaths		8.2 (7.2, 9.9)	2.3 (1.7, 3.2)	5.3 (4.6, 6.6)	6362 (5646, 7690)	1657 (1249, 2287)	8019 (6888, 9977)			
Total based on registrations		5.7 (4.0, 8.5)	2.2 (1.4, 3.2)	4.0 (2.7, 5.9)				10063 (6941 14822)	3616 (2370, 5413)	13679 (9310, 20235)
Total cancers in GB in ages 15+					77 912	72 212	150 124	175 399	168 184	343 583

Abbreviations: GB=Great Britain; NHL=non-Hodgkin's lymphoma; NMSC=non-melanoma skin cancer; STS=soft tissue sarcoma.

Totals do not always sum across rows due to rounding error.

Confidence intervals not estimated for cancers attributed to ionising radiation, as they are not yet available for the excess relative risk models used (UNSCEAR, 2006).

a AF applicable to all leukaemias.

b Includes cases described as due to paraoccupational or environmental exposure to asbestos.

c Taken as equal to attributable deaths for this short survival cancer.

d Based on registrations.

**Table 2:** Cancer registrations in 2004 attributable to occupation by exposure and cancer sites with at least 15 total attributable registrations

	**Cancer site^a^**																	
**Carcinogen or occupation**	**Bladder**	**Brain**	**Breast**	**Cervix**	**Larynx**	**Leukaemia**	**Lung**	**Mesothelioma**	**Nasopharynx**	**NMSC**	**NHL**	**Oesophagus**	**Ovary**	**Sinonasal**	**STS**	**Stomach**	**Other sites**	**Total registrations**
Asbestos					8		2223	1937								47		4216
Shift work (including flight personnel)			1969															1969
Mineral oils	296						470			902				63				1730
Solar radiation										1541								1541
Silica							907											907
Diesel engine exhaust	106						695											801
PAHs from coal tars and pitches										545								545
Painters	71						282									5		359
TCDD (dioxins)							215				74				27			316
Environmental tobacco smoke (non-smokers)							284											284
Radon							209											209
Welders							175											175
Tetrachloroethylene				18							17	130						164
Arsenic							129											129
Strong inorganic-acid mists containing sulphuric acid					46		76											122
Chromium VI							67							22				89
Non-arsenical insecticides		11				19					33						MM (10)	73
Cobalt							73											73
Inorganic lead		2					42									23		67
Aromatic amines	66																	66
Hairdressers and barbers	15										14		33					63
Soots												60						60
Wood dust									14					39				54
Leather dust														31				31
Steel foundry workers							29											29
Formaldehyde						12			1					1				14
PAHs	7						4											11
Nickel							10							0				10
Cadmium							9											9
Beryllium							7											7
Trichloroethylene											3						Kidney (3) Liver (2)	7
Benzene						7												7
UV radiation (welders only)																	Melanoma (eye) (6)	6
Rubber industry					3											1		4
Ionising radiation						1	2										Bone (0) Liver (0) Thyroid (1)	4
Vinyl chloride																	Liver (3)	3
Tin miners							2											2
1,3-Butadiene						0											LH (1)	1
Acrylamide																	Pancreas (1)	1
Ethylene oxide						1												1
Petroleum refining		0																0
Total registrations attributable to occupation	550	14	1969	18	56	40	5447	1937	16	2928	140	188	33	133	27	158	26	13 679
Total registrations in GB (2004)^b^	9878	3933	43202	2612	2112	5202	37378	2037	189	67 220	8236	7498	6197	378	1063	7970	22 034^c^	339 156^d^

Abbreviations: GB=Great Britain; LH=lymphohaematopoietic cancers; MM=multiple myeloma; NHL=non-Hodgkin's lymphoma; NMSC=non-melanoma skin cancer; PAH=polycyclic aromatic hydrocarbon; STS=soft tissue sarcoma; TCDD=2,3,7,8-Tetrachlorodibenzodioxin; UV=ultraviolet.

a Blank cells indicate that attributable cancer registrations were not estimated for this occupational exposure. Zero represents an estimate of less than 0.5.

b Registrations aged 25+ years for solid tumours, aged 15–84 years for haematopoietic neoplasms for men and 15–79 years for haematopoietic neoplasms for women; figures for mesothelioma based on deaths.

c Includes bone, kidney, liver, melanoma (eye), multiple myeloma, pancreas, thyroid.

d All malignant neoplasms.

**Table 3:** Industry sectors and occupations with an estimate of a total of at least 50 attributable registrations, by cancer site and occupational exposure

	**Attributable registrations**				
**Industry sector^a^**	**Men**	**Women**	**Total**	**Exposure^b^**	**Cancer site^c^**
Construction (CAREX F)	4752	64	4816	**Ar**, **Asb**, *Ca*, Ch, Co, **DEE**, **ETS**, F, *N*, **Pb**, PAH, R, **Si**, **Sr**, **Tet**, **W**	Brain, **bladder**, larynx, **lung**, **mesothelioma**, nasopharynx, **oesophagus**, **NMSC**, **sinonasal**, **stomach**
Roofers, road surfacers, roadmen, paviors (Construction)^d^	538	3	541	**PAHc**	**NMSC**
Painters and decorators (Construction)^d^	331	3	334	**Painting**	**Bladder**, **lung**, **stomach**
**Total construction**	**5621**	**71**	**5692**		
Shift work (including flight personnel)	1	1970	1971	**Shift work, IR**	**Breast**
Metal workers^d^	1081	169	1,250	**Mineral oils**	**Bladder**, **lung**, **NMSC**, **sinonasal**
Personal and household services	274	530	804	**AA**, **Asb**, Bz, *Ca*, Ch, **DEE**, **ETS**, F, **HB**, mineral oils, PAH, *Pb*, R, **soots**, **Sr**, **Tet**, Tri	**Bladder**, **cervix**, kidney, leukaemia, **lung**, **mesothelioma**, **NHL**, **NMSC**, **Oesophagus**, **Ovary**, sinonasal, stomach
Land transport	463	42	505	**Asb**, Bz, Ch, **DEE**, ETS, IR, PAH, *Pb*, R, *S*, Si, Sr, Tet, W	**Bladder**, **leukaemia**, **lung**, **mesothelioma**, NMSC, oesophagus, stomach
Mining	285	17	302	**Asb**, **DEE**, IR, N, PAH, Pb, R, **Si**, **Sr**, Tin mining	Bladder, **lung**, **mesothelioma**, NMSC, stomach
Printing, publishing and allied industries	235	51	286	Ch, Co, DEE, **mineral oils**, PAH, Pb, R, Sr, Tet, W	**Lung**, NMSC, oesophagus, sinonasal
Public administration and defence	239	34	273	DEE, **ETS**, PAH, *Pb*, **R**, **Sr**	**Lung**, **NMSC**
Wholesale and retail trade and restaurants and hotels	110	159	269	**Asb**, Bz, *Ca*, *Ch*, D, DEE, **ETS**, PAH, *Pb*, **R**, Sr	Leukaemia, **lung**, **mesothelioma**, NHL, STS, NMSC
Farming	180	39	220	**D**, ETS, **NAP**, R, **Sr**	Brain, **leukaemia**, **lung**, MM, **NHL**, **NMSC**, STS
Manufacture of instruments, photographic and optical goods	204	2	206	Be, Ch, Co, DEE, **mineral oils**, R, *Si*, VCM, W	**Bladder**, lung, NMSC, sinonasal
Manufacture of transport equipment	170	18	188	Ar, **Asb**, Be, **Ch**, Co, DEE, mineral oils, N, PAH, R, **Si**, SIA, Sr, Tet, Tri, W	Cervix, kidney, larynx, **lung**, **mesothelioma**, NHL, NMSC, oesophagus, sinonasal, stomach
Welders^d^	165	16	181	UV, **Welding fumes**	**Lung**, melanoma (eye)
Non-ferrous metal basic industries	125	34	159	**Ar**, Bz, Ca, Ch, Co, DEE, D, F, N, PAH, Pb, R, Si, **SIA**, Sr, Tet, VCM, W	Bladder, larynx, liver, leukaemia, **lung**, **NHL**, NMSC, sinonasal, stomach, STS
Iron and steel basic industries	119	16	135	**AA**, Bz, Ch, DEE, **D**, F, Mineral oils, PAH, Pb, R, SIA, Sr, **steel foundry workers**, Tet, W	**Bladder**, larynx, **lung**, **NHL**, oesophagus, sinonasal, stomach, **STS**
Manufacture of other chemical products	110	13	123	Ac, Ar, **Asb**, Bz, 1-3B, Ch, DEE, EO, F, Pb, R, Si, **SIA**, VCM, W	Larynx, liver, **lung**, **mesothelioma**, sinonasal, stomach
Manufacture of industrial chemicals	107	14	121	AA, Ac, Ar, **Asb**, Bz, 1-3B, Ca, Ch, Co, **D**, DEE, F, NAP, PAH, Pb, R, **SIA**, Si, VCM, W	Bladder, larynx, leukaemia, liver, LH, **lung**, **mesothelioma**, MM, NHL, pancreas, sinonasal, stomach, STS
Manufacture of machinery except electrical	81	30	111	Be, **Ch**, Co, DEE, F, IR, Mineral oils, PAH, R, **Si**, **SIA**, **Tet**, Tri, W	Cervix, kidney, larynx, **lung**, NHL, **oesophagus**, sinonasal
Painters, not construction^d^	77	25	102	**Painting including spray painting**	**Bladder**, **lung**, **stomach**
Manufacture of pottery, china and earthenware	65	33	98	Ch, Co, **D**, PAH, R, **Si**, SIA, **Sr**, Tet	**Lung**, NHL, **NMSC**, STS
Sanitary and similar services	39	56	95	Ar, **Asb**, Bz, *Ca*, Ch, Co, DEE, ETS, IR, PAH, *Pb*, R, **Sr**, *Tri*, W	**Lung**, mesothelioma, **NMSC**
Manufacture of fabricated metal products, except machinery and equipment	69	25	94	Be, **Ch**, **Co**, DEE, F, N, PAH, R, **Si**, **SIA**, Sr, **Tet**, Tri, W	Cervix, larynx, **lung**, NHL, NMSC, **oesophagus**, sinonasal
Electricity, gas and steam	69	20	89	Ar, **Asb**, Be, *Ca*, Ch, Co, DEE, IR, *N*, PAH, *Pb*, R, Si, **Sr**, Tet, W	**Lung**, **mesothelioma**, **NMSC**, oesophagus
Manufacture of other non-metallic mineral products	57	17	75	Ar, Ch, Co, DEE, F, **D**, PAH, R, **Si**, Sr, VCM, W	Bladder, **lung**, NHL, NMSC
Manufacture of wood and wood and cork products, except furniture	55	14	69	**Ar**, Ch, Co, DEE, **D**, F, PAH, R, Sr, **W**	Leukaemia, **lung**, NHL, nasopharynx, NMSC, sinonasal, STS
Recreational and cultural services	32	37	69	Ar, ETS, *F*, R, **Sr**, *Tri*	**Lung**, **NMSC**
Manufacture of glass and glass products	49	15	64	Ar, Be, Ch, Co, DEE, **D**, F, PAH, R, **Si**, Sr, Tet, W	**Lung**, **NHL**, NMSC, STS
Financing, insurance, real estate and business services	31	32	63	**ETS**, **R**, Sr	**Lung**, NMSC
Manufacture of paper and paper products	56	5	61	**Asb**, 1-3B, Ch, Co, DEE, D, PAH, R, SIA, Tet, W	Larynx, **lung**, **mesothelioma**, oesophagus

Abbreviations: AA=aromatic amines (bladder); Ac=acrylamide (pancreas); Ar=arsenic (lung); Asb=asbestos (larynx, lung, mesothelioma, stomach); Be=beryllium (lung); Bz=benzene (leukaemia); Ca=cadmium (lung); Ch=chromium IV (lung, sinonasal); Co=cobalt (lung); D=dioxins (lung, NHL, STS); DEE=diesel engine exhaust (lung, bladder); EO=ethylene oxide (leukaemia); ETS=environmental tobacco smoke (lung); F=formaldehyde (sinonasal, leukaemia); HB=hairdressers and barbers (bladder, NHL, ovary); IR=ionising radiation (bone, leukaemia, liver, lung, thyroid); LD=leather sust (sinonasal); MWF=metal working fluids (bladder, NMSC, sinonasal); N=nickel (lung, sinonasal); NAP=non-arsenical pesticides (brain, leukaemia, MM, NHL); PAH=polycyclic aromatic hydrocarbons (lung, bladder); PAHc=coal tars and pitches (NMSC); Pb=lead (brain, lung, stomach); R=radon (lung); Si=silica (lung); Sr=solar radiation (NMSC); SIA=strong inorganic acid mists (larynx, lung); Tet=tetrachloroethylene (cervix, NHL, oesophagus); Tri=trichloroethylene (liver, kidney, NHL); VCM=vinyl chloride monomer (liver); W=wood dust (nasopharynx, sinonasal); 1-3B=1,3-butadiene (leukaemia).

a ISIC Rev. code (CAREX data) unless otherwise stated.

b Exposures in italics were at low or background level only with a relative risk estimated as 1 and therefore zero attributable cancers; exposures in bold account for at least 10 attributable registrations in men plus women.

c Cancer sites in bold account for at least 10 attributable registrations in men plus women, other sites contribute at least 1.

d Labour Force Survey (LFS), 1979 or 1991.

**Table A1:** Employment level adjustment and turnover factors used in the calculation of AF

	**Main industry sector**	**Adjustment factor for change in employment levels^a^**	**Turnover per year (%)**
*Men*			
A–B	Agriculture, hunting and forestry; fishing	1	7
C–E	Mining and quarrying, electricity, gas and water; manufacturing industry	1.4	9
F	Construction	1	12
G–Q	Service industries	0.9	11
	Total	1	10
			
*Women*			
A–B	Agriculture, hunting and forestry; fishing	0.75	10
C–E	Mining and quarrying, electricity, gas and water; manufacturing industry	1.5	14
F	Construction	0.67	15
G–Q	Service industries	0.8	15
	Total	0.9	14

a Applied to CAREX data for the solid tumour REP only. Exposed numbers are obtained for a mid-point year in the REP where national employment data sources have been used (the LFS or CoE).

## Statistical Appendix

### Formulae used in the estimation of AF

1. Levin's equation

where RR=relative risk, Pr(E)=proportion of the population exposed.

A common denominator is used across exposure levels and industries for each exposure.

2. Miettinen's equation

where Pr(E∣D)=proportion of cases exposed (E=exposed, D=case).

3. Turnover equation to estimate numbers ever employed during the REP

where *N*_e(REP)_=numbers ever employed in the REP; *n*_0_=numbers employed in the exposed job/industry at a mid-point in the REP; TO=staff turnover per year; *R*=retirement age (65 for men, 60 for women); *l*_(adj15)*i*_=the proportion of survivors to age *i* of those alive at age 15 (from GB life tables); *a* to *b*=age range achieved by the original cohort members by the target year (2004) (e.g., 65–100 for the solid tumour REP); *c* to *d*=age range achieved by the turnover recruited cohort members by the target year (25–64 for the solid tumour REP); age(*u*) and age(*l*)=upper and lower recruitment age limits (24 and 15). The equation can be represented as a single factor behaving as a multiplier for *n*_0_, calculated by setting *n*_0_ to 1 in the above equation, so that the factor varies only with TO, see Table A1.

4. Equation to estimate the proportion of the population exposed

where *N*_p(REP)_=numbers ever of working age during the REP from population estimates for the relevant age cohorts in the target year.

5. Equation for combining AFs where exposed populations overlap but are independent and risk estimates are assumed to be multiplicative:

for the *k* exposures in the set.

## Supplementary Material

Supplementary Table A1
