# Supplementary material for: Occupation and cancer in Britain
Source: Br J Cancer. 2010 Apr 27;102(9):1428–37. doi: 10.1038/sj.bjc.6605637 (PMC2865752; doi:10.1038/sj.bjc.6605637)
Supplement: Supplementary Table A1 [file 6605637x1.doc]

**Table A1** Estimated attributable fractions, deaths and registrations by cancer site in 2005 (deaths) and 2004 (registrations) for agents and occupations classified as IARC group 1 with ‘strong’ evidence of carcinogenicity in humans

| **Cancer Site** |  | **Attributable Fraction (%)**  **(95% Confidence Interval)** | | | **Attributable Numbers**  **(95% Confidence Interval)** | | | | | |
| --- | --- | --- | --- | --- | --- | --- | --- | --- | --- | --- |
|  |  |  | | | **Deaths (2005)** | | | **Registrations (2004)** | | |
|  | **ICD-10 code** | **Male** | **Female** | **Total (Based on Deaths)** | **Male** | **Female** | **Total** | **Male** | **Female** | **Total** |
| Bladder | C67 | 0.8 (0.7,3.0) | 0.6 (0.5,2.9) | 0.7 (0.6,2.8) | 24 (20, 91) | 10 (9, 39) | 34 (29, 130) | 55 (47, 211) | 18 (16, 70) | 73 (63, 280) |
| Bone | C40-C41 | 0 | 0 | 0 | 0 | 0 | 0 | 0 | 0 | 0 |
| Brain | C70-C72 | 0 | 0 | 0 | 0 | 0 | 0 | 0 | 0 | 0 |
| Breast | C50 |  | 0 | 0 |  | 0 | 0 |  | 0 | 0 |
| Cervix | C53 | 0 | 0 | 0 | 0 | 0 | 0 | 0 | 0 | 0 |
| Kidney | C64-C66, C68 | 0 | 0 | 0 | 0 | 0 | 0 | 0 | 0 | 0 |
| Larynx | C32 | 2.3 (0.8,5.1) | 1.5 (0.5, 3.4) | 2.1 (0.8, 4.8) | 14 (5, 31) | 2 (1, 5) | 16 (6, 37) | 40 (15, 89) | 6 (2, 12) | 46 (17, 102) |
| Leukaemia ^a^ | C91-C95 | 0.1 (0, 2.0) | 0.2 (0.1, 3.9) | 0.2 (0, 2.6) | 3 (0, 40) | 2 (0, 36) | 5 (1, 75) | 5 (1, 70) | 4 (0, 55) | 8 (1, 124) |
| Liver | C22 | 0.2 (0.1,0.3) | 0.1 (0, 0.1) | 0.1 (0.1, 0.2) | 2 (1, 4) | 1 (0, 2) | 3 (1, 6 ) | 2 (1, 4) | 1 (0, 2) | 3 (2, 6) |
| Lung | C33-C34 | 17.6 (15.5, 19.4) | 4.4 (3.5, 5.4) | 12.0 (10.2, 13.9) | 3347 (2945,3687) | 599 (527,660) | 3946 (3472,4346) | 3853 (3390,4244) | 673 (592, 741) | 4526 (3982, 4985) |
| Lympho-haematopoietic | C81-C96 | 0 | 0 | 0 | 0 | 0 | 0 | 0 | 0 | 0 |
| Melanoma (eye) | C69 | 0 | 0 | 0 | 0 | 0 | 0 | 0 | 0 | 0 |
| Mesothelioma | C45 | 97.0 (96.0, 98.0)^b^ | 82.5 (75.0, 90.0) ^b^ | 94.9 (93.0, 96.9) ^b^ | 1699 (1681, 1717) | 238 (216, 260) | 1937 (1898, 1976) | 1699 (1681, 1717)^c^ | 238 (216, 260) ^c^ | 1937 (1898, 1976) ^c^ |
| Multiple Myeloma | C90 | 0 | 0 | 0 | 0 | 0 | 0 | 0 | 0 | 0 |
| Nasopharynx | C11 | 0 | 0 | 0 | 0 | 0 | 0 | 0 | 0 | 0 |
| NHL | C82-C85 | 0 | 0 | 0 | 0 | 0 | 0 | 0 | 0 | 0 |
| NMSC^d^ | C44 | 7.1 (1.3, 15.1) | 1.1 (0.0, 2.9) | 4.6 (0.8, 10.0) | 21 (4, 44) | 2 (0, 6) | 23 (4, 50) | 2576 (481, 5475) | 352 (0, 900) | 2928 (481, 6375) |
| Oesophagus | C15 | 0 | 0 | 0 | 0 | 0 | 0 | 0 | 0 | 0 |
| Ovary | C56 | 0 | 0 | 0 | 0 | 0 | 0 | 0 | 0 | 0 |
| Pancreas | C25 | 0 | 0 | 0 | 0 | 0 | 0 | 0 | 0 | 0 |
| Sinonasal | C30-C31 | 21.1 (11.8, 34.7) | 13.6 (8.2, 22.5) | 17.7 (10.2, 29.2) | 13 (7, 22) | 7 (4, 12) | 20 (11, 34) | 46 (26, 76) | 22 (12, 36) | 68 (38, 112) |
| STS | C49 | 0 | 0 | 0 | 0 | 0 | 0 | 0 | 0 | 0 |
| Stomach | C16 | 0 | 0 | 0 | 0 | 0 | 0 | 0 | 0 | 0 |
| Thyroid | C73 | 0.12 | 0.02 | 0.05 | 0 | 0 | 0 | 1 | 0 | 1 |
| Total Based on deaths |  | 6.6 (6.0, 7.2) | 1.2 (1.0, 1.4) | 4.0 (3.6, 4.4) | 5123 (4665, 5635) | 862 (758, 1019) | 5986 (5415, 6612) |  |  |  |
| Total Based on registrations |  | 4.7 (3.2, 6.8) | 2.0 (1.3, 2.8) | 3.4 (2.3, 4.8) |  |  |  | 8277 (5642, 11886) | 1313 (839, 2075) | 9590 (6482, 13962) |
| Total cancers in GB in ages 15+ |  |  |  |  | 77912 | 72212 | 150124 | 175399 | 168184 | 343583 |

^a^ AF applicable to all leukaemias

^b^ Includes cases described as due to paraoccupational or environmental exposure to asbestos.

^c^ Taken as equal to attributable deaths for this short survival cancer.

^d^ Based on registrations.

Totals do not always sum across rows due to rounding error

Confidence Intervals not estimated for cancers attributed to ionizing radiation, as they are not yet available for the excess relative risk models used (UNSCEAR 2006)
